# Disseminated Intravascular Coagulation as a Possible Cause of Acute Coronary Stent Thrombosis: A Case Report and Literature Review

**DOI:** 10.1155/2012/178260

**Published:** 2012-12-17

**Authors:** Syed Amer, Ali Shafiq, Waqas Qureshi, Mohammed Muqeetadnan, Syed Hassan

**Affiliations:** ^1^Department of Medicine, Henry Ford Hospital, Detroit, MI 48202, USA; ^2^Department of Medicine, University of Oklahoma Health Sciences Center, Oklahoma City, OK 73104, USA

## Abstract

Disseminated intravascular coagulation (DIC), as a cause of acute coronary stent thrombosis, has not yet been reported to our knowledge. We report a case of 64-year-old male, who presented with non-ST-segment elevation myocardial infarction (NSTEMI). Coronary angiography revealed right coronary artery (RCA) stenosis and a drug eluting stent was deployed. Fifteen hours following the intervention, the patient developed an inferior wall ST elevation myocardial infarction. Repeat cardiac catheterization showed an acute in-stent thrombosis. Following thrombectomy, another stent was placed. The patient noted to have an acute drop in platelet count following the second intervention. Two hours following repeat intervention, the patient again developed chest pain and EKG showed recurrent ST-segment elevations in leads II, III, and aVF. Prior to repeat cardiac catheterization, the patient became unresponsive and developed cardiogenic shock. The patient was resuscitated and intubated, and repeat catheterization showed complete stent thrombosis. Intracoronary tissue plasminogen activator (tPA) was given. The platelet count further dropped. Additional studies confirmed the diagnosis of DIC. No further cardiac catheterization was done at this point. The patient then later had a cardiac arrest and unfortunately cardiopulmonary resuscitation could not revive him. Amongst the etiologies of acute stent thrombosis, DIC was deemed a possible cause.

## 1. Introduction

Percutaneous coronary intervention (PCI) is widely performed for the treatment of obstructive coronary artery disease (CAD) with good results [1, 2]. Acute stent thrombosis is an unusual complication after PCI [3]. Among the etiologies of acute stent thrombosis, DIC has not been reported before. We describe a case of the patient who developed DIC after suffering myocardial infarction and subsequently had recurrent coronary stent thrombosis.

## 2. Case Description

**Figure 1:**
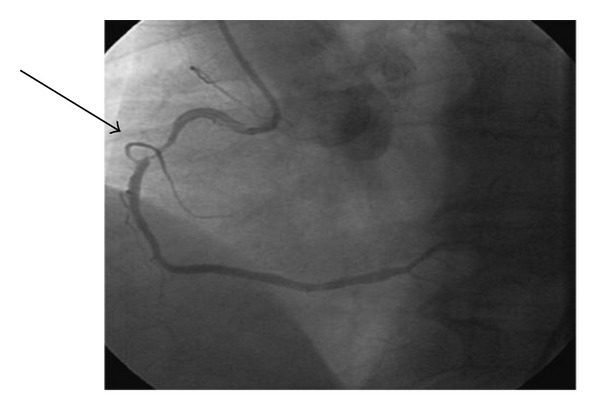
RCA angiogram showing >90% stenosis.

**Figure 2:**
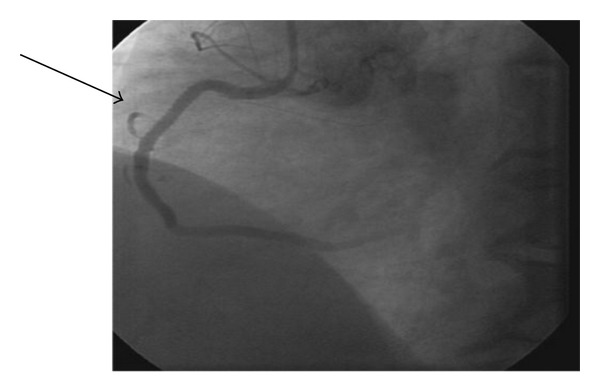
Post RCA stent residual showing 0% stenosis.

A 64-year-old Caucasian male presented with left arm pain and numbness for 1 day. The patient was not on any medications and had no family history of coronary artery disease or sudden death. Physical examination was unremarkable for any significant abnormalities. Electrocardiogram (ECG) showed normal sinus rhythm and T wave inversions in leads II, III, and aVF. Chest X-ray was negative for any acute process. Patient was started on aspirin 325 mg along with intravenous heparin, nitroglycerin, and eptifibitide. Initial troponin-I level was at 0.47 ng/mL which later increased to 2.28 ng/mL, and a decision was made to perform left heart catheterization. RCA angiogram showed >90% stenosis (Figure 1) and a drug eluting stent was deployed with poststent residual 0% stenosis and Thrombolysis in Myocardial Infarction (TIMI) III flow (Figure 2). The patient was started on clopidogrel after the procedure at a loading dose of 600 mg.

The patient developed left sided chest pain 15 hours following the initial intervention and ECG revealed an ST segment elevation in leads II, III, and aVF. Repeat cardiac catheterization showed an acute thrombosis of the initially placed stent. Thrombectomy was performed with Export catheter and another drug eluting stent was placed. 

**Figure 3:**
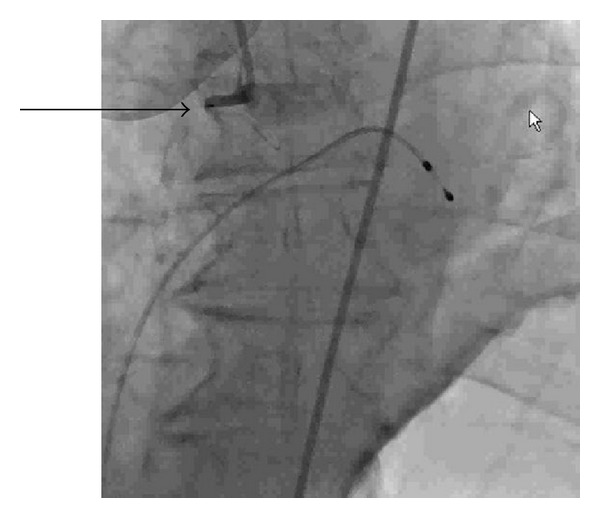
RCA revealing 100% thrombosis of the recently placed stent.

There was an acute drop in platelet count to 74,000/*μ*L from 145,000/*μ*L following the second intervention. Heparin-induced thrombocytopenia (HIT) was suspected. IV heparin was stopped and argatroban was started. Heparin platelet factor 4 antibodies results were 0.419 OD (mildly abnormal), which were not significant to confirm HIT. Two hours later, the patient again complained of chest pain and EKG showed recurrent ST-segment elevations in the inferior leads. Prior to repeat cardiac catheterization, the patient became unresponsive, hypotensive and developed ventricular tachycardia. The patient was cardioverted and started on vasopressors. After resuscitation a transvenous pacemaker and an intra-aortic balloon pump were placed. Repeat angiogram of the RCA revealed 100% thrombosis of the recently placed stent (Figure 3).

Intra-arterial TPA and thrombectomy were performed which allowed partial restoration of the flow. Clopidogrel was replaced with prasugrel.

Further workup showed a rise in creatinine to 2.02 mg/dL, and a drop in platelet count to 46,000/mcL and skin showed generalized petechiae. The patient started bleeding from his nasogastric tube and ultrasound of the abdomen revealed free intraperitoneal fluid. A workup for DIC was performed which showed an international normalized ratio (INR) of 7, partial thromboplastin time (PTT) of 205, D dimer: 1400, and fibrinogen of 84 mg/mL. Fresh frozen plasma and blood transfusion were given. Patient's echocardiogram showed right ventricular dilatation with hypokinesis, consistent with right ventricular infarction. The patient continued to deteriorate clinically despite all aggressive therapy. There was a continuous rise in troponin I levels and persisted ST segment elevation. No further cardiac catheterization was done despite an ST elevation in inferior wall leads. The patient later had a cardiac arrest and cardiopulmonary resuscitation could not revive him.

## 3. Discussion

Acute coronary stent thrombosis remains a worrisome complication after PCI. There are many risk factors and causes associated with this problem. The patient in this case presented with NSTEMI, underwent PCI with stent placement, and developed recurrent acute stent thrombosis despite multiple revascularization attempts. A workup was done to determine the possible etiology for these events. A final diagnosis was not made with certainty for the cause of the thrombotic events. Amongst the differential diagnoses, DIC was also considered as a possible cause. We present a review of literature related to the definition and causes of acute stent thrombosis and discuss DIC as a possible etiological factor.

Stent thrombosis has been classified in previous studies as acute (intraprocedural or within 24 hours of the procedure), subacute (from 24 h to 30 days), late (30 days to 1 year), or very late (1 year). Acute and subacute ST were also defined as early ST. General risk factors associated with stent thrombosis include malignant disease, old age, black race, and diabetes mellitus. [4–6]. Predictors of stent thrombosis include bifurcation lesions, under sizing, uncovered dissection, and suboptimal procedural result (TIMI flow grade < 3 after PCI). None of these factors were present in our patient. Patients with low left ventricular ejection fraction, use of drug eluting stents, and total number of stents are also important predictors for early stent thrombosis [7]. Heparin-induced thrombocytopenia has been reported to cause acute stent thrombosis [8] and was suspected in our patient. However the heparin platelet factor 4 antibodies were 0.419 OD (mildly abnormal) which were not consistent with a diagnosis of HIT [9]. Clopidogrel and aspirin resistance have been shown to be associated with stent thrombosis [10, 11]. Our patient suffered from very early stent thrombosis despite therapy with antiplatelet agents (aspirin, clopidogrel, and eptifibitide). Prasugrel was also substituted later in the course of his management. His clinical deterioration despite use of multiple medications suggested that these were unlikely the dominant cause of the patient's cascade of events. Our patient developed DIC after his multiple coronary interventions. DIC as a cause of early stent thrombosis has never been reported and was considered as a possible cause in the absence of other etiologies.

There is no direct evidence of the association of DIC with coronary stent thrombosis. However indirect evidence suggests that DIC may have a role in these measures. DIC has been shown to be involved in the thrombosis of coronary vessels [12] and severe cardiac dysfunction as a result of the thrombotic occlusive events [13]. The body's natural anticoagulants, namely, protein C and antithrombin-III have been found to be at low levels in patients with DIC [14–16]. This predisposes the blood vessels to increased thrombosis. This has also been suggested by previous literature, for instance, protein C deficiency has been shown to cause thrombosis of the mesenteric artery [17], coronary artery, and jugular vein [18]. We reviewed a case report in which AT-III deficiency was shown to be associated with recurrent coronary in-stent thrombosis in an emergent PCI [19]. These examples are suggestive that it is a likely possibility that DIC may lead to recurrent coronary stent thrombosis in the absence of other obvious causes. 

After repetitive NSTEMIs our patient developed ecchymosis, purpura, and intraperitoneal bleeding. His lab work showed severe thrombocytopenia, low fibrinogen, and elevated PT and PTT levels. The clinical scenario suggested a diagnosis of DIC. We excluded common causes of DIC such as sepsis, trauma, and malignancy. Clopidogrel has been shown to cause DIC and was considered as a possible etiological factor. We also found that acute myocardial infarction has been reported to cause DIC [20]. As our patient suffered from multiple myocardial insults, AMI was deemed the likely cause of the DIC in this scenario.

## 4. Conclusion

A patient undergoing acute stent thrombosis in emergent percutaneous interventions may have fatal results. We want to emphasize that DIC should be considered as one of the etiological factors in cases of acute stent thrombosis.

## References

[1] Serruys PW, De Jaegere P, Kiemeneij F (1994). A comparison of balloon-expandable-stent implantation with balloon angioplasty in patients with coronary artery disease. *The New England Journal of Medicine*.

[2] Fischman DL, Leon MB, Baim DS (1994). A randomized comparison of coronary-stent placement and balloon angioplasty in the treatment of coronary artery disease. *The New England Journal of Medicine*.

[3] Shiraishi J, Kohno Y, Sawada T (2007). In-hospital outcomes of primary percutaneous coronary interventions performed at hospitals with and without on-site coronary artery bypass graft surgery. *Circulation Journal*.

[4] Kuchulakanti PK, Chu WW, Torguson R (2006). Correlates and long-term outcomes of angiographically proven stent thrombosis with sirolimus- and paclitaxel-eluting stents. *Circulation*.

[5] Park SH, Hong GR, Seo HS, Tahk SJ (2005). Stent thrombosis after successful drug-eluting stent implantation. *Korean Circulation Journal*.

[6] Kim S, Jeong M, Sim D (2009). Very late thrombosis of a drug-eluting stent after discontinuation of dual antiplatelet therapy in a patient treated with both drug-eluting and bare-metal stents. *Korean Circulation Journal*.

[7] van Werkum JW, Heestermans AA, Zomer AC (2009). Predictors of coronary stent thrombosis. The dutch stent thrombosis registry. *Journal of the American College of Cardiology*.

[8] Al-Lamee RK, Gerber RT, Kooner JS (2010). Heparin-induced thrombocytopenia (HIT) as an unusual cause of acute stent thrombosis. *European Heart Journal*.

[9] Warkentin TE, Sheppard JI, Moore JC, Sigouin CS, Kelton JG (2008). Quantitative interpretation of optical density measurements using PF4-dependent enzyme-immunoassays. *Journal of Thrombosis and Haemostasis*.

[10] Kim S, Jeong M, Kim H, Bae S, Ryu K, Cho K (2009). Acute and subacute stent thrombosis in a patient with clopidogrel resistance: a case report. *Korean Circulation Journal*.

[11] Ruef J, Kranzhöfer R (2006). Coronary stent thrombosis related to aspirin resistance: what are the underlying mechanisms?. *Journal of Interventional Cardiology*.

[12] Sugiura M, Hiraoka K, Ohkawa S (1977). A clinicopathological study on cardiac lesions in 64 cases of disseminated intravascular coagulation. *Japanese Heart Journal*.

[13] Ueda K, Sugiura M, Ohkawa S (1981). Disseminated intravascular coagulation in the aged complicated by acute myocardial infarction. *Japanese Journal of Medicine*.

[14] Takahashi H, Takakuwa E, Yoshino N (1985). Protein C levels in disseminated intravascular coagulation and thrombotic thrombocytopenic purpura: its correlation with other coagulation parameters. *Thrombosis and Haemostasis*.

[15] Marlar RA, Endres-Brooks J, Miller C (1985). Serial studies of protein C and its plasma inhibitor in patients with disseminated intravascular coagulation. *Blood*.

[16] Kogan AE, Strukova SM (1990). Protein C decreases in experimental DIC in rats. *Thrombosis Research*.

[17] Onwuanyi A, Sachdeva R, Hamiram K, Islam M, Parris R (2001). Multiple aortic thrombi associated with protein C and S deficiency. *Mayo Clinic Proceedings*.

[18] Cakir O, Ayyildiz O, Oruc A, Eren N (2002). A young adult with coronary artery and jugular vein thrombosis: a case report of combined protein S and protein C deficiency. *Heart and Vessels*.

[19] Kaku B, Katsuda S, Taguchi T, Nitta Y, Hiraiwa Y (2009). A case of acute myocardial infarction with repetitive stent thrombosis during emergent percutaneous coronary intervention: transient decrease in antithrombin III activity and heparin resistance. *International Heart Journal*.

[20] Thomson FJ, Benbow EW, McMahon RFT, Cheshire CM (1991). Pulmonary infarction, myocardial infarction, and acute disseminated intravascular coagulation. *Journal of Clinical Pathology*.

